# Discovery of structural alterations in solid tumor oligodendroglioma by single molecule analysis

**DOI:** 10.1186/1471-2164-14-505

**Published:** 2013-07-26

**Authors:** Mohana Ray, Steve Goldstein, Shiguo Zhou, Konstantinos Potamousis, Deepayan Sarkar, Michael A Newton, Elizabeth Esterberg, Christina Kendziorski, Oliver Bogler, David C Schwartz

**Affiliations:** 1 Laboratory for Molecular and Computational Genomics, Department of Chemistry, Laboratory of Genetics, UW Biotechnology Center, University of Wisconsin-Madison, Madison, WI 53706, USA; 2 Theoretical Statistics and Mathematics Unit, Indian Statistical Institute, Delhi, India; 3 Department of Biostatistics and Medical Informatics, University of Wisconsin-Madison, Madison, WI 53706, USA; 4 Department of Statistics, University of Wisconsin-Madison, Madison, WI 53706, USA; 5 M.D. Anderson Cancer Center, University of Texas, Houston, TX 77030, USA

**Keywords:** Cancer genomics, Oligodendroglioma, Single molecule, Optical mapping, Structural variation, Mutation

## Abstract

**Background:**

Solid tumors present a panoply of genomic alterations, from single base changes to the gain or loss of entire chromosomes. Although aberrations at the two extremes of this spectrum are readily defined, comprehensive discernment of the complex and disperse mutational spectrum of cancer genomes remains a significant challenge for current genome analysis platforms. In this context, high throughput, single molecule platforms like Optical Mapping offer a unique perspective.

**Results:**

Using measurements from large ensembles of individual DNA molecules, we have discovered genomic structural alterations in the solid tumor oligodendroglioma. Over a thousand structural variants were identified in each tumor sample, without any prior hypotheses, and often in genomic regions deemed intractable by other technologies. These findings were then validated by comprehensive comparisons to variants reported in external and internal databases**,** and by selected experimental corroborations. Alterations range in size from under 5 kb to hundreds of kilobases, and comprise insertions, deletions, inversions and compound events. Candidate mutations were scored at sub-genic resolution and unambiguously reveal structural details at aberrant loci.

**Conclusions:**

The Optical Mapping system provides a rich description of the complex genomes of solid tumors, including sequence level aberrations, structural alterations and copy number variants that power generation of functional hypotheses for oligodendroglioma genetics.

## Background

Cancer is fundamentally a disease of genomic origin. Alterations in genes and regulatory elements critical to cell cycle control lead to uncontrolled cell growth and proliferation, the common signature of all cancers. Such events can cause amplification or mutational activation of oncogenes [1,2], deletion or mutation deactivation of tumor suppressor genes [3,4], orientation of genes with incorrect regulatory regions [5], gene fusion products [6], etc. As cancers evolve, they accumulate a cascade of mutations, ranging in size from a single nucleotide change to the gain or loss of entire chromosomes [7]. Coupled with the subclonal heterogeneity that is a hallmark of solid tumors [8-10], obtaining a complete portrait of the genetic landscape of human cancer remains a significant challenge.

Synergy between revolutionary genomic tools and advances in high-throughput computing has facilitated the development of a number of methods for detecting mutations. Chromosome banding and spectral karyotyping (SKY) [11] are low-resolution techniques used to detect large-scale chromosomal features. However, obtaining metaphase spreads for performing a karyotype is often difficult, especially when working with solid tumor biopsies and paraffin embedded, formalin fixed tissue. Fluorescence in situ hybridization (FISH) and its variants are a family of molecular cytogenetic techniques developed to correlate specific sequences to cytogenetic observations [12]. FISH offers higher resolution (compared to SKY) and has the advantage of not requiring metaphase spreads, but is limited by the fact that it requires a prior hypotheses about the locus of interest, making it unsuitable for discovery based research. Hybridization based microarray approaches, like SNP microarrays and array comparative genome hybridization (CGH), have been extensively used to detect large scale amplifications and deletions in tumor genomes [13-15], but are unable to detect changes where there is no net gain or loss of DNA, such as inversions and balanced translocations, which have been shown to be an important mechanism for oncogenic transformation [16-18]. Moreover, microarrays do not offer structural information, necessitating follow-up experiments to identify the breakpoints and sequence context of the aberration. Microarrays are also restricted to regions of the genome amenable to unique probe design, which precludes repeat-rich regions and novel insertions that are hotbeds of variation and mutation [19-22]. Most commercial microarrays (except custom designed, high-density arrays) lose sensitivity below ~50 kb, and variants, particularly insertions, in this size range have remained largely unexplored, especially in cancer genomes [23].

The advent of massively parallel, short read DNA sequencing- the ‘second generation’ sequencing technologies, and their application to cancer has also accelerated the pace of mutation discovery. Initially applied to targeted subsets of the genome, such as specific gene families (e.g.: all protein kinases, or ‘kinome’) [24-27], or all the coding sequences (the exome) [28-33], second-generation sequencing is increasingly being used to interrogate whole cancer genomes [34-40]. In theory, second-generation sequencing of whole genomes has the ability to discern the full range of genomic alterations. In practice, however, more than 90% of events discovered by these platforms are less than 1 kb, and are biased towards deletions rather than insertions [23,41]. Second-generation sequencing instruments typically generate shorter reads with higher error rates from relatively short insert libraries, which present a significant computational and bioinformatic challenge in alignment and assembly [42]. Read-pair mapping approaches have successfully identified point mutations and indels in cancer [36,38-40], but are limited by the insert size of the DNA library to detecting base substitutions and small indels [43] and are often confounded by repetitive regions of the genome. Further, accurate prediction of the exact breakpoints of an aberration depends on very tight size distribution of the DNA library, which can make library construction difficult [44]. Whole genome sequencing followed by *de novo* assembly might mitigate some of these issues, but current assembly algorithms tend to collapse homologous sequences, and consequently dramatically under-represent repeats and segmental duplications that are known to be critical mediators of genomic rearrangement [42].

There remains a pressing need for discovery-based systems that can provide a scalable, comprehensive view of the cancer genome in its entirety. In this study, we present Optical Mapping as one such system. Optical Mapping creates high-resolution ordered restriction maps of whole genomes through the analysis of ensembles of single molecule restriction maps. It has previously been used to map the genomes of microbes [45-48], plants [49-52] and mammals [53-57]. However, this is the first time it has been employed to analyze the genome of a solid tumor. Optical Mapping offers several unique advantages towards assembling the complex structure of a cancer genome. Genomic DNA isolated directly from cells is analyzed, thereby obviating any bias introduced by amplification or cloning steps. Moreover, because the DNA is of high-molecular weight (300 kb - >500 kb), segmental duplications and other repeat-rich regions of the genome are revealed, and additionally, the structure and long-range context of any aberration are determined. Since the restriction maps are made from single DNA molecules, Optical Mapping effectively pieces together heterogeneous alterations, which is especially important for tumor genome analysis, as we demonstrate in oligodendroglioma.

Oligodendrogliomas are frontal lobe tumors that are thought to arise from oligodendrocytes, supporting brain cells which provide myelination for neurons [58,59]. The concerted loss of heterozygosity (LOH) of chromosome arms 1p and 19q, observed in 50-70% of patients, is a molecular signature of this malignancy [60]. The remarkably high prevalence of this molecular marker suggests that these regions harbor one or more tumor suppressor genes that might play an important role in the development of the tumor. Allelic losses of 1p/19q have been correlated with positive response to chemo- and radiotherapy and prolonged survival for patients with oligodendroglioma [61]. However, it remains unclear whether LOH of 1p/19q is a prognostic biomarker for a more indolent tumor subtype that has fewer unfavorable mutations overall, rather than predictive of treatment sensitivity [62,63]. In fact, studies have shown that 1p/19q codeleted tumors have slower growth rates and are more responsive to treatment than tumors without the codeletion [64,65]. In order to explore each of these possibilities, Optical Mapping was used to create physical maps from two individual oligodendroglioma tumor biopsies for the purpose of identifying and characterizing structural changes on a whole genome basis.

## Results and discussion

### Optical map construction

**Figure 1:**
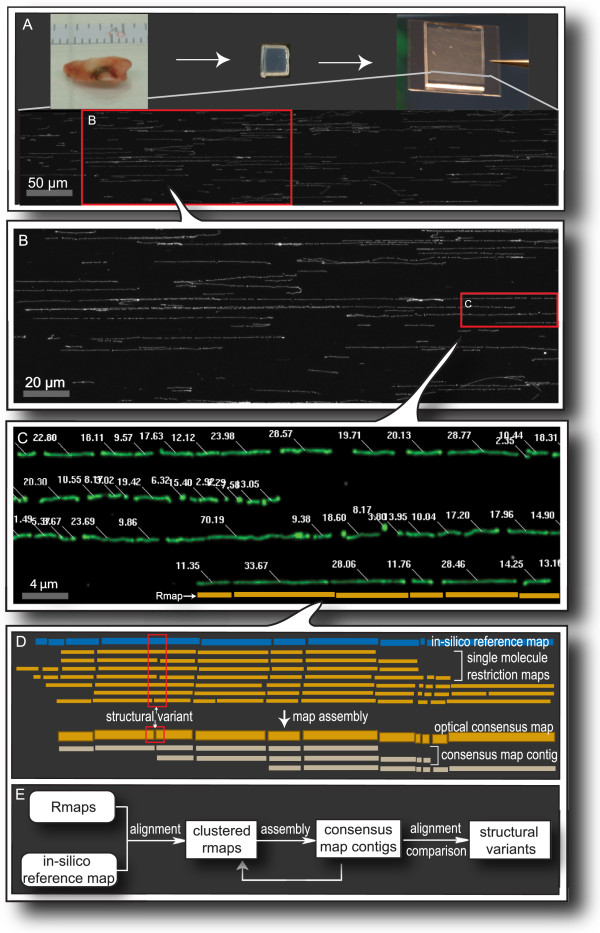
**An overview of the Optical Mapping system. A**: Single cells, obtained from a slice of the tumor biopsy, are purified by a percoll density gradient, then mixed with agarose and allowed to solidify in a mold, forming rectangular inserts. Prior to mapping, cells are lysed within the insert, the DNA electrophoretically extracted, elongated and immobilized on an Optical Mapping surface by means of capillary flow through a microchannel device. The lower half of panel A is a representative image of properly elongated DNA (long, white horizontal lines) after surface digestion, stained with YOYO-1. Microchannels are 100 μm wide as indicated by the scale bar (grey bar). **B**: Enlarged image of surface-bound genomic DNA, digested with SwaI, showing discrete restriction fragments separated by gaps. **C**: Automated machine vision detects DNA molecules (pseudocolored green), and calculates the mass of each fragment (white numbers), creating ordered restriction maps (Rmaps) from single DNA molecules (yellow bars). **D** and **E**: Strategy for constructing a genome-wide optical map starting from single molecule Rmaps. Rmaps are first clustered on a restriction map generated *in-silico* from the reference sequence of the human genome by pairwise alignment. Then consensus optical map contigs are constructed by *de novo* assembly of the Rmaps from a given window. Finally, the consensus map contigs are aligned back to the reference map, and differences are identified.

We used the Optical Mapping (OM) system to explore the genomic landscape of a solid tumor. Optical Mapping creates high-resolution physical maps of genomes through the analysis of ensembles of single molecule ordered restriction maps. The tumor biopsies were disaggregated into single cells, then run through a Percoll gradient to enrich for cancer cells (methods). High molecular weight genomic DNA was extracted directly from these cells, stretched and immobilized in regular arrays on positively charged glass surfaces using a microfluidic device (Figure 1A, details in methods) [66]. After deposition, the DNA was digested with the restriction enzyme SwaI. The surface-bound restriction fragments remained in register, and were stained with a fluorescent dye and imaged by automated fluorescent microscopy (Figure 1B). Dedicated machine vision software calculated the size, in kilobase pairs (kb), of each fragment based on measurements of integrated fluorescent intensity, resulting in the high throughput, massively parallel generation of ordered restriction maps, or ‘Rmaps’, from individual genomic DNA molecules (Figure 1C) [66]. The oligodendroglioma datasets comprise close to 700,000 such Rmaps, with an average size of greater than 400 kb (Additional file 1). The relative order and distance between successive restriction fragments in a single molecule optical map can be used to determine the precise location in the genome that gave rise to that molecule, by means of pair-wise alignment against an *in-silico* restriction map [67]. The HF087 tumor dataset comprised 235,026 aligned Rmaps, corresponding to 36.92 fold coverage of the human genome, while the HF1551 dataset comprised 167,012 aligned maps, representing 25.36 fold coverage of the human genome (Additional file 1). In the absence of a karyotype, our assessment of ploidy is based on optical map coverage and Affymetrix array analysis. Both these platforms, discussed in detail in subsequent sections, calculate chromosome copy number relative to normal, diploid genomes, and are in agreement that neither tumor sample is polyploid. They do, however, display aneuploidy, due to allelic losses of specific chromosomes/chromosome arms (1p, 19q, 13 in HF087 and 1p, 19q, 14, 21 in HF1551), so if anything the coverage is likely to be higher than what we reported.

The Rmaps that cluster together upon pair-wise alignment were then assembled into consensus optical maps and analyzed for presence of structural variants using the bioinformatics pipeline described in [56]. The final consensus map contigs span 96.73% and 93.92% of the human genome for tumors HF087 and HF1551, respectively.

### Optical map coverage analysis

#### ***Discernment of copy number variants***

**Figure 2:**
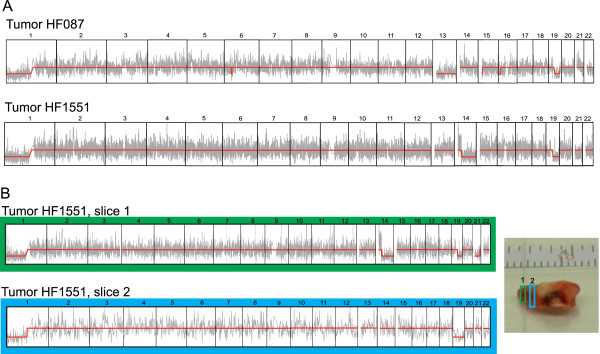
**Intra-tumor heterogeneity. A**: Copy number profiles, inferred from analysis of optical map coverage of tumor HF087 and HF1551. For each panel, the x-axis is co-ordinates of the human genome (chromosome numbers are indicated at the top), and y-axis is counts of Rmaps that align to a particular genomic interval. The grey curve plots the observed number of counts in an interval, and the red line indicates the sequence of underlying copy number states (also called the Viterbi path). **B**: Copy number analysis of tumor HF1551 by slice. Slice 1 (green) has LOH of chromosomes 1p, 19q, 14 and 21, while slice 2 (blue) has losses of chromosomes 1p and 19 only.

Copy number was inferred from aligned coverage of Rmaps, prior to assembly, in a manner analogous to read-depth based methods for detecting copy number variants from second generation sequencing data (methods). Briefly, Rmaps were aligned to the *in silico* reference map, and then partitioned into discrete windows spanning each chromosome. These alignments were then compared to alignments of a reference data set (comprising of a number of normal genomes) that was used to ‘normalize’ the observed coverage. This was necessary because the number of Rmaps that align to a particular region of the genome depends, in part, on the density of restriction sites in that region, which varies from chromosome to chromosome (ranging from a low of 2.5 cuts/100 kb on chromosome 22 to a high of 9.25 cuts/100 kb on chromosome 4). A Hidden Markov Model (HMM) was then fitted to this data, and copy number changes were detected (Figure 2A) [68,69]. Optical map coverage analysis confirmed the allelic loss of chromosome arms 1p and 19q in HF087 and HF1551. The breakpoints appear to be very close to the centromere, consistent with the proposed mechanism of an unbalanced reciprocal translocation mediating the LOH event [70,71]. Additionally, coverage analysis also detected allelic loss of chromosome 13 (HF087), 14 and 21 (HF1551), which are known to be rarer events associated with oligodendroglioma.

#### ***Solid tumor heterogeneity***

The genome wide optical map of HF1551 was created using DNA from two adjacent slices of the tumor: 446,933 (~55%) Rmaps originated from slice 1 and 202,974 (~45%) Rmaps from slice 2 (Figure 2). Interestingly, when the Rmaps were partitioned according to the slice they originated from, and coverage analysis was performed separately, unique copy number profiles were obtained for each slice. In addition to allelic losses of 1p and 19q, slice 1 also had LOH of chromosomes 14 and 21, while slice 2 had evidence of LOH of 19p (Figure 2B). Solid tumors are dynamic aggregates of continually evolving subclones, resulting in spatial and temporal genetic heterogeneity. Our findings suggest that the tumor slices used for Optical Mapping evolved from distinct cancer cell clones, and is congruent with recent evidence of branched evolutionary tumor growth [72-74]. Although assembly of whole genome maps on a per slice basis was not feasible due to insufficient depth of coverage, our results establishes proof-of-principle of Optical Mapping to interrogate tumor heterogeneity.

### Discovery of optical structural alterations

**Figure 3:**
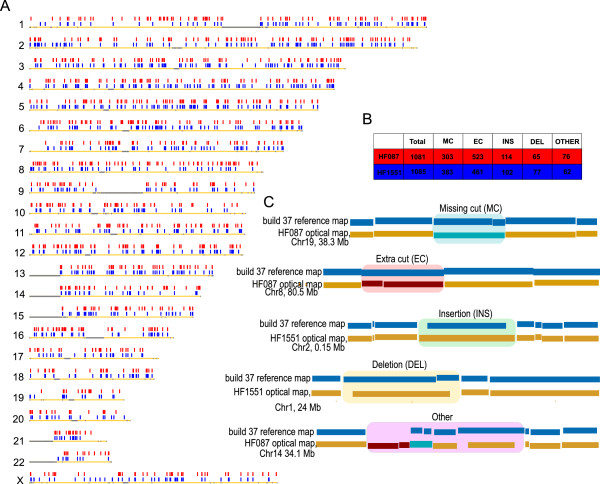
**Genome-wide distribution of optical structural alterations (OSAs) detected in oligodendroglioma. A**: Horizontal yellow bars, numbered on the left, represent human chromosomes (heterochromatic regions are in grey). Tick marks depict locations of structural variants from HF087 (red) and HF1551 (blue). **B**: The total number of events detected in each tumor sample, also broken down by category. **C**: An example of each class of variant is shown in the inset figure.

The optical consensus maps generated by map assembler were aligned to the *in silico* restriction map (generated from build 35 human reference sequence), and by comparison of the order and sizing of the 219,224 restriction fragments (fragments smaller than 0.4 kb in size were merged) between the experimental and the reference map. Such comparisons revealed structural variants in the experimental genome that were classified as four types: extra cuts (EC), where the optical consensus map displays a restriction site that was not predicted by the reference sequence; missing cuts (MC), where a cut that was predicted was not observed in the experimental map; insertions (INS), where the size of a fragment in the consensus map was significantly larger than its counterpart in the reference map; deletions (DEL), where a fragment in the experimental map was smaller than the corresponding reference fragment (or missing altogether); and finally, complex events (OTHER) involving multiple cut or size differences (methods). Approximately a third of the ECs and MCs represent small indels that are below the resolution of Optical Mapping (~3 kb) [56]. Figure 3C shows an example of each class of variant detected by Optical Mapping.

At first glance, it might appear that any one of these variants could be attributed to errors inherent in Optical Mapping. For instance, a missing cut could be due to incomplete digestion, an extra cut could result from spurious cutting by the restriction enzyme, or physical breakage of the DNA molecule, and uneven staining could lead to inaccurate estimation of fragment size. However, the high throughput advantage of Optical Mapping allows us to distinguish such random errors from legitimate genomic events. Any alteration in the optical consensus map was supported by multiple single molecule maps (Rmaps), each representing an independent observation at that locus. The Optical Mapping error models estimated the statistical significance of each structural variant, after taking into account the quality and quantity of the data [56].

**Figure 4:**
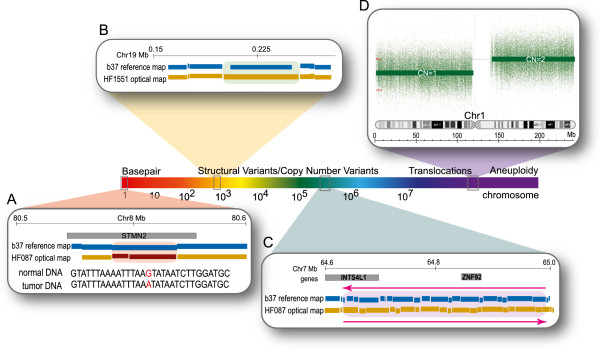
**Spectrum of genomic alterations in oligodendroglioma. A**: ~800 single base alterations were found in each tumor, like the G > A transition in the STMN2 gene shown. **B**: A ~8 kb insertion from tumor HF1551. 179 such indels were detected per sample. **C**: A 352 kb inversion that disrupts pseudogene INTS4L1 and encompasses the zinc-finger transcription factor ZNF92. **D**: Loss of one copy of chromosome 1p, a hallmark of oligodendroglioma.

**Figure 5:**
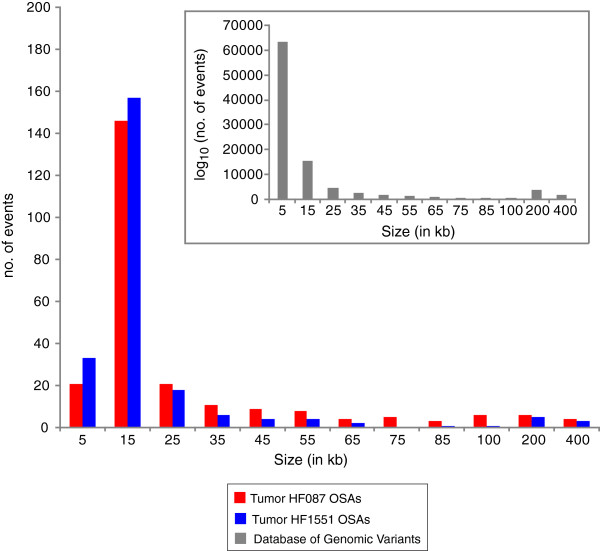
**Size distribution of indels found by Optical Mapping.** Histogram of indel sizes detected by Optical Mapping. The x-axis is size of indel in kilobase pairs, and the y-axis is number of events. For comparison, a similar graph is shown (inset) of the distribution of indel sizes from the Database of Genomic Variants.

A total of 1081 and 1085 differences were detected in HF087 and HF1551 respectively (Figure 3A and B). The distribution of structural variants across the genome is uniform and the pattern is similar for both tumors (Figure 3A). Variants range in size from single base differences to complex genomic events spanning hundreds of kilobases (Figure 4). Approximately 800 single base changes were detected in each tumor, including point mutations (such as the example depicted in Figure 4A), polymorphic SNPs where only one allele has a SwaI restriction site (referred to as snip-SNPs [75]), and small indels that create or remove a SwaI cut site but are below the detection limit of Optical Mapping. 179 indels with a median size of 6.6 kb were detected in each sample (Figure 5). For comparison, the median size of indels reported in the Database of Genomic Variants is 2.3 kb (Figure 5, inset). ~70 complex events were found in each tumor, including known polymorphic loci such as the major histocompatibility complex (MHC), giving us confidence that these results are not spurious. Optical Mapping also discerns balanced genomic events, where there is no net gain or loss of genomic sequence. A putative inversion spanning 352 kb of chromosome 7 was observed in HF087 which appears to disrupt the ZNF92 gene (Figure 4C). Finally, the largest events detected by Optical Mapping include gains or losses of entire arms of chromosomes, for example, the allelic loss of chromosome 1 illustrated in Figure 4D, and discussed in detail previously. Intersection counts with genes, segmental duplications, published SNPs (dbSNP build 135, http://www.ncbi.nlm.nih.gov/projects/SNP/) and published structural variants (Database of Genomic Variants, November 2010 release) are shown in Additional file 2. Comprehensive breakdown of the overlaps are shown in Additional file 3.

Optical Mapping provides a comprehensive description of the vast and complex landscape of cancer genomes. The ability to study the genome in its entirety, including non-genic or repetitive regions using a single technology minimizes ascertainment bias. As detailed in subsequent sections, it is employed to generate a list of candidate cancer genes that is not hypothesis-limited, and elucidate their structure at sub-genic resolution.

### Validation of copy number and structural variants

#### ***Experimental validation: SNP array***

The Affymetrix Genome Wide Human SNP 6.0 Array, which has probes for detection of both SNPs and copy number variants (CNVs), was used to validate our findings.

Both platforms concurred on the LOH of chromosomes 1p, 19q and 13, but allelic loss of chromosome 14 in HF1551 was not detected by the Affymetrix array. The copy number profile generated by running the HMM algorithm on the maps from the first slice of tumor HF1551 was similar to that from the array, which suggests that the DNA originated from tumor sections that were in closer proximity.

Many of the SNP probes on the Affymetrix chip correspond to SwaI snip-SNPs. Hence, the array data was used to validate ECs and MCs. We observed 100% (62/62 in HF087, 44/44 in HF1551) concordance between the SNP genotype and the SwaI cut pattern at all overlapping cut differences in both tumors (Additional file 4).

The copy number variants detected by the array were also compared to Optical Mapping indels. Signal intensities from the chip were normalized by global median scaling, and copy number was assessed using several different algorithms (methods), relative to a reference model file generated from the 270 HapMap samples. Though the resolution of array CGH is much lower than Optical Mapping, we were able to validate 24 structural variants in tumor HF087 and 16 in tumor HF1551 (Additional file 5).

#### ***Experimental validation: PCR***

The nature of many of the structural variants, being within repetitive portions of the genome, but detected by Optical Mapping unfortunately precludes their comprehensive validation by simple PCR techniques. Accordingly, we selected two variants that were amenable to PCR and overlapped genes that may offer insights into the chemo- and radio-sensitivity of oligodendroglioma. These loci were then PCR amplified, cloned and sequenced (methods).

**Figure 6:**
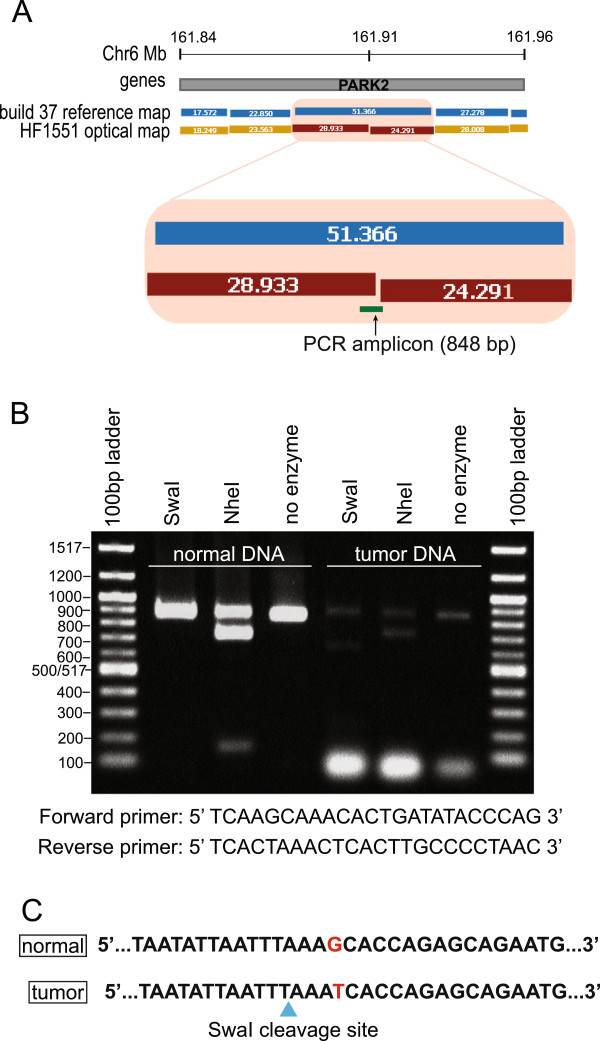
**Experimental validation of PARK2 mutation by PCR-sequencing. A**: EC in PARK2 gene on chromosome 6 of HF1551. The enlarged figure shows the position of the PCR amplicon. **B**: Restriction digest of the PCR amplicon. The undigested amplicon is 848 bp. Digestion with SwaI restriction enzyme is expected to yield two fragments of 577 bp and 271 bp (based on the location of the EC in the optical map). An addition digestion was performed with NheI enzyme to ensure the correct amplicon was being analyzed. The expected sizes of the NheI fragments are 700 bp and 148 bp. **C**: Sequence of the PCR amplicon showing the G > T transversion that creates a new SwaI cut site.

The optical map shows an EC in the PARK2 gene in HF1551 (Figure 6A). PARK2 is a putative tumor suppressor, and mutations in this gene have been reported in multiple cancer types (detailed in ‘candidate mutations’ section of this document). An 848 bp amplicon spanning the predicted location of the EC was obtained (Figure 6B), and Sanger sequencing proved that a G to T transversion resulted in the creation of a new SwaI restriction site (Figure 6C).

**Figure 7:**
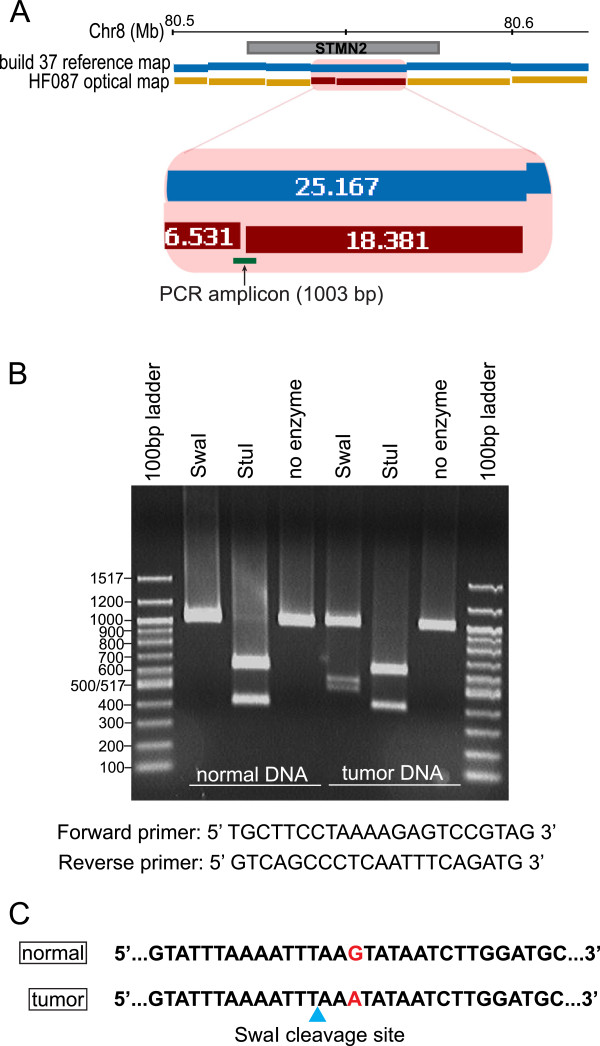
**Experimental validation of an EC in the STMN2 gene by PCR-sequencing. A**: EC in STMN2 gene on chromosome 8 of HF087. The enlarged figure shows the position of the PCR amplicon. **B**: Restriction digest of the PCR amplicon. The undigested amplicon is 1003 bp. Digestion with SwaI restriction enzyme is expected to yield two fragments of 519 bp and 484 bp (based on the location of the EC in the optical map). An addition digestion was performed with NheI enzyme to ensure the correct amplicon was being analyzed. The expected sizes of the NheI fragments are 616 bp and 387 bp. **C**: Sequence of the PCR amplicon showing the G > A transition that creates a new SwaI cut site (blue triangle).

We also validated an EC in tumor HF087 that occurred in the STMN2 gene (Figure 7A). As discussed in subsequent sections, STMN2 regulates microtubule dynamics and is believed to be a target of beta-catenin/TCF signalling. We amplified a 1003 bp region around the putative mutation (Figure 7B), and were able to validate the alteration via sequencing (Figure 7C).

#### ***Comparative validation***

We also validated our findings by comparing them to two sources- Optical Mapping data from several normal genomes, and publicly available SNP and structural variant data. First, oligodendroglioma structural variants were compared against structural variants found by Optical Mapping of 6 other normal human genomes by our laboratory. This internal database includes: (three lymphoblast-derived cell lines and a complete hydatiform mole (dbVar study ID nstd49) [56], a lymphocyte-derived cell line (unpublished) and an early passage human embryonic stem cell line [55]). 80%-90% of oligodendroglioma variants were also detected in at least one of the normal human genomes (Additional file 6), suggesting that such loci are polymorphic, and affirming the veracity of our findings. Then, oligodendroglioma structural variants were compared against variants in the Database of Genomic Variants (DGV). The DGV is an extensive catalogue of structural variation in normal humans, currently holding 101,923 events detected by a variety of platforms. We observed the greatest concordance with variants found by fosmid-end sequencing (~15%) and high density oligonucleotide array CGH (~10%) (Additional file 6). Finally, cut differences detected by Optical Mapping were compared to published SNPs. Detailed breakdown of these intersections are shown in Additional file 3; parameters for comparisons are described in the Methods section.

### Candidate mutations

#### ***Separation of mutational and polymorphic OSAs***

The ultimate goal of our mapping efforts was to identify genes or genomic elements that maybe important to the biology of oligodendroglioma, with the caveat that such ‘candidates’ represent hypotheses requiring rigorous testing to establish their functional role in tumorigenesis. Distinguishing between structural polymorphisms and somatically acquired mutations is a key step towards accomplishing this goal. Unfortunately, matched normal DNA from the individuals whose tumors were optically mapped was not available. Instead, we adopted a stringent filtering scheme to remove putative polymorphisms and enrich for somatic mutations, based on comparisons to internal and publicly available data (described above). Parameters for these comparisons were determined based on the Optical Mapping error model and designed to be extremely parsimonious (methods). As a result of these operations, we arrived at a total of 21 somatic mutations (5 genes) in HF087 and 73 somatic mutations (21 genes) in HF1551.Since two mutations are seen in both tumors, 24 unique candidate cancer genes were identified in oligodendroglioma (Table 1). A few interesting candidate genes will be discussed in the next section.

**Table 1:** Candidate cancer genes identified in oligodendroglioma

**Gene symbol**	**Gene name**	**Entrez gene ID**	**Location**	**Tumor sample**
ALMS1	Alstrom syndrome 1	7840	chr2,73612885,73837046	HF1551
APPL1	adaptor protein, phosphotyrosine interaction, PH domain and leucine zipper containing 1	26060	chr3,57261764,57307498	HF1551
ARHGAP10	Rho GTPase activating protein 10	79658	chr4,148653452,148993927	HF1551
CCDC91	coiled-coil domain containing 91	55297	chr12,28410132,28703099	HF1551
CECR2	cat eye syndrome chromosome region, candidate 2	27443	chr22,17956627,18033845	HF1551
DIAPH2	diaphanous homolog 2 (Drosophila)	1730	chrX,95939661,96724837	HF087
EFHC2	EF-hand domain (C-terminal) containing 2	80258	chrX,44007127,44202923	HF1551
EIF1	eukaryotic translation initiation factor 1	10209	chr17,39845126,39847898	HF1551
LASS3	LAG1 homolog, ceramide synthase 3	204219	chr15,100940599,101084925	HF1551
LOC339166	uncharacterized RNA coding gene	339166	chr17,5675553,5834016	HF1551
LRRN2	leucine rich repeat neuronal 2	10446	chr1,204586302,204654481	HF1551
MYOF	myoferlin	26509	chr10,95066185,95242074	HF1551
NPAS3	neuronal PAS domain protein 3	64067	chr14,33408458,34273382	HF087, HF1551
OSBPL3	oxysterol binding protein-like 3	26031	chr7,24836163,25019760	HF087, HF1551
PARK2	Parkinson disease (autosomal recessive, juvenile) 2, parkin	5071	chr6,161768589,163148834	HF1551
PAX7	paired box 7	5081	chr1,18957499,19075360	HF1551
PHLDB2	pleckstrin homology-like domain, family B, member 2	90102	chr3,111451326,111695364	HF1551
PLEKHM3	pleckstrin homology domain containing, family M, member 3	389072	chr2,208686011,208890284	HF1551
PRKG1	protein kinase, cGMP-dependent, type I	5592	chr10,52750910,54058110	HF1551
SIPA1L3	signal-induced proliferation-associated 1 like 3	23094	chr19,38397867,38699008	HF087
STMN2	stathmin-like 2	11075	chr8,80523048,80578410	HF087
TACC2	transforming, acidic coiled-coil containing protein 2	10579	chr10,123748688,124014057	HF1551
TCEB3	transcription elongation factor B (SIII), polypeptide 3	6924	chr1,24069855,24088549	HF1551
ZFYVE26	zinc finger, FYVE domain containing 26	23503	chr14,68213236,68283306	HF1551

#### ***Candidates common to both HF087 and HF1551***

**Figure 8:**
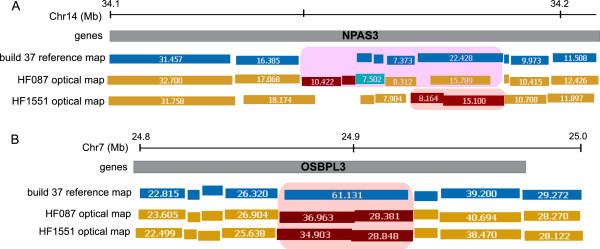
**Candidate genes harboring OSAs in both HF087 and HF1551. A**: NPAS3 which bears a complex alteration in HF087 and an EC in HF1551. **B**: OSBPL3 which harbors cut differences in both tumor samples.

Two candidate genes, NPAS3 and OSBPL3, harbored mutations in both tumor samples (Figure 8). NPAS3 (neuronal PAS domain protein 3) shows a complex event accompanied by a ~7 kb gain in the HF087 optical map, and a missing cut in the HF1551 optical map (Figure 8A). This neuronally expressed basic helix-loop-helix transcription factor has been implicated in schizophrenia [76,77]and bipolar disorder [77], and is frequently deleted or inactivated in many cancers. Recently, it has been demonstrated that NPAS3 exhibits features of a tumor suppressor which drives late progression of malignant astrocytomas, and is a negative prognostic marker for survival [78].

Both tumor optical maps display cut differences in the OSBPL3 (oxysterol binding protein like-3) gene (Figure 8B). This gene plays a vital role in cell adhesion, cytoskeletal organization and lipid metabolism [79-81]. It is highly expressed in B-cell associated malignancies [82,83], where it is one of the common sites of retroviral integration [84]. An independent study that used exon sequencing to study oligodendroglioma also found somatic mutations in OSBPL3 [30].

#### ***Candidates observed in either HF087 or HF1551***

In the HF1551 optical map, we observe a point mutation that creates a SwaI restriction site in the PARK2 gene (Figure 6). This gene encodes an E3 ubiquitin ligase, called *Parkin* that catalyzes the ubiquitination of a variety of target proteins for proteasome mediated degradation. Germline mutations in PARK2 have long been known to cause autosomal recessive juvenile Parkinson’s disease [85-87]. More recently, PARK2 has been identified as a tumor suppressor gene in Glioblastoma multiforme, breast, ovary, lung, colorectal and liver cancers [28,88-94]. It encompasses most of FRA6E, the third most active common fragile site in the human genome [95], and shares the characteristics of other tumor suppressors such as FHIT and WWOX, that also occur in fragile sites. PARK2 is frequently deleted or inactivated in cancer cell lines and primary tumors [88,92], and concomitantly, *Parkin* expression is either significantly diminished or absent [89,92]. Unlike classical tumor suppressors where biallelic inactivation is necessary for oncogenesis, heterozygous mutations in PARK2 are sufficient to confer a growth advantage during tumor development [88,92]. Restoring *Parkin* expression in *Parkin*-deficient cell lines reduces their profileration *in vitro*[92], while injection of *Parkin*-deficient cells into immunocompromised mice generate tumors *in vivo*[91]. Interestingly, PARK2 also mediates chemosensitivity in breast cancer *via* microtubule dependent mechanism [93,96-98].

STMN2 (stathmin-like 2) is another interesting candidate gene. We observe a point mutation in this gene in tumor HF087 (Figure 7). STMN2 is a neuron specific member of the stathmin family of small regulatory phosphoproteins which control cell profileration and differentiation [99]. It is up-regulated in liver cancer and has been identified as a target of β-catenin/TCF-mediated transcription [100]. STMN2 sequesters soluble tubulin, forming a ternary complex, inhibits microtubule assembly and induces their disassembly [101]. Its highly similar, but more well-studied paralog STMN1, located on chromosome 1p, is known to sensitize cells to anti-microtubule drugs in glioma [102,103], breast [104,105] and prostate cancer [106].In light of recent studies demonstrating the synergistic epistasis between paralogous genes involved in essential cellular functions and its therapeutic implications [107,108], we speculate that STMN1 and STMN2 might be functionally redundant, and inactivation of STMN2 might, in part, explain the treatment sensitivity of oligodendroglioma.

In the HF1551 optical map, we see an extra cut in the gene ZFYVE26 (zinc finger, FYVE domain containing 26). Spastizin, the zinc finger protein encoded by ZFYVE26, causes the neurological disorder hereditary spastic paraplegia [109]. This gene binds to the tumor suppressor Beclin-1 and regulates cytokinesis [110,111], and is recurrently mutated in breast cancer [29].

**Figure 9:**
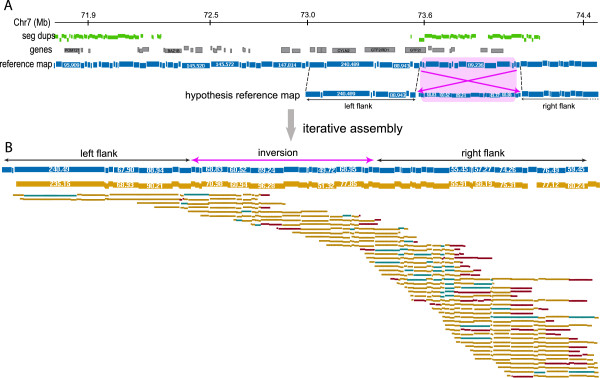
**Strategy for assembling the ~500 kb inversion in the Williams-Beuren region in HF1551. A**: Construction of the modified, ‘hypothesis’ reference map for directed assembly. The map has a ~500 kb inversion in the center, flanked on either side by 500 kb of sequence that agrees with the reference map. **B**: After 8 iterations of map assembly, an optical consensus map is obtained that spans the hypothesized reference, and has multiple Rmaps that bridges across both left and right breakpoints.

We detected a 485 kb inversion on 7q11.23 in tumor HF1551. Hemizygous deletions spanning a 1.5-1.8 MB region of this locus cause the neurodevelopmental disorder, Williams-Beuren (WB) syndrome [112]. However, inversion of this region is polymorphic, and is present in ~6% of the general population, and in ~25% of transmitting parents in WB families [113-115]. Given the disparity in size between the aberration detected by OM and reported instances of the WB inversion, it is possible that the event we observe arose de novo and is distinct from the ‘canonical’ inversion. To test this hypothesis, we ran several targeted assemblies on the WB region. The general strategy for this approach was to modify the reference map in-silico to reflect our hypothesized structure (Figure 9A), and then use the iterative assembly framework described earlier to pull out individual restriction maps and generate an optical consensus map (methods). Since our map assembly pipeline was designed to provide the single most conservative answer, this approach is helpful in detecting large-scale aberrations that are significantly different from the reference sequence. Of the eight modified reference maps we started with, the one that reflected the canonical WB inversion/deletion did not grow, while the one that reflected the 485 kb event successfully generated a consensus map that spanned it and flanked contiguous regions on chromosome 7 (Figure 9B). The optical consensus map also closes the putative sequence gap immediately to the right of the inversion, and in fact, approximately half of the sequence gaps in the reference genome (NCBI, build 35) are spanned by optical consensus maps. The inversion encompasses the genes GTF2IRD2, PMS2P5, WBSCR16, GTF2IRD2B and NCF1, and its breakpoints appear to disrupt the genes GTF2I and STAG3L2. In the absence of matched normal DNA, it is impossible to ascertain if the inversion we detected was inherited through the germline or somatically acquired, however, this is the first report, to the best of our knowledge, of inversions in the WB region in the context of cancer.

#### ***Candidates on 1p or 19q***

The concerted loss of chromosome arms 1p and 19q is a hallmark of oligodendroglioma. Seen in 50%-70% of tumors, it is believed that these regions harbor one or more tumor suppressor genes that play an important role in the development of this cancer. Hence, somatic mutations on these chromosome arms are particularly interesting. We found putative mutations on 2 genes residing on chromosome 1p (TCEB3, PAX7) and 1 gene on 19q (SIPA1L3). The roles of these genes in normal and disease states, and the structural variants we found in them are discussed briefly in the subsequent section.

We observe a 6.3 kb deletion that potentially ablates the first exon of TCEB3 in tumor HF1551. TCEB3 (transcription elongation factor B, polypeptide 3) encodes the transcriptionally active subunit of the mammalian elongin complex [116,117]. This elongation factor stimulates the rate of transcription by suppressing the transient pausing of RNA polymerase II on the DNA template [118]. TCEB3 is part of a multi-protein complex that functions as an elongin-based ubiquitin ligase [119], similar to the Von Hippel-Lindau (VHL) tumor suppressor complex, by mediating DNA damage induced ubiquitination and degradation of polymerase II [120].

Tumor HF1551 also has an insertion in the 1p-encoded gene PAX7 (paired box 7). The PAX genes encode a family of transcription factors that control development within the neural, myogenic and lymphoid lineages [121]. PAX7, in particular, is essential for survival, proliferation and migration of myogenic progenitor cells [122], and cell fate decisions in the developing nervous system [123]. PAX7 is the target of a recurrent gene fusion with the forkhead protein FKHR/FOXO1 that is found in ~15% of patients with alveolar rhabdomyosarcoma [121,124]. The fusion transcript is much more abundant and transcriptionally active than wild type PAX7 [125], suggesting that the deregulation of PAX7 downstream target genes contribute to tumorigenesis.

In the HF087 optical map, we observe a missing cut in the gene SIPA1L3 (signal induced proliferation associated 1 like 3) which is located on the long arm of chromosome 19. This gene encodes a Ras specific GTPase activating protein that is found at epithelial junctional complexes. These complexes play a crucial role in mechanical adhesion between epithelial cells to form cellular sheets and in the organization of actin cytoskeleton [126]. Somatic mutations in SIPA1l3 have been discovered in cancers of the brain [127,128], prostate [129], breast [24], ovary [32], pancreas [130], colon [24], skin [131] and hematopoietic system [34], but a cohesive picture of the functional role that this gene plays in these diverse cancer types is yet to emerge.

Taken together, the candidate genes discovered by Optical Mapping point to critical roles of transcriptional control and cytoskeletal organisation in the etiology of oligodendroglioma.

#### ***Non-genic candidates***

Protein coding sequences comprise less than 2% of the human genome. The vast non-coding portion of the genome, once believed to be ‘junk DNA’, is rife with functional elements that orchestrate the gene expression program of cells. Recent evidence from the ENCODE (Encyclopedia of DNA Elements) consortium indicates that as much as 80.4% of the human genome encodes a defined product (for instance, a non-coding RNA) or displays a reproducible biochemical signature (for instance, a specific chromatin structure) [132]. Such signatures, either alone or in combinations, mark genomic sequences with important functions, such as promoters, enhancers, insulators and silencers [133]. The ENCODE data sheds some light on possible functional roles of Optical Mapping candidates that are not located within genes. A number of these candidates are actively transcribed, for instance an EC on chromosome 5 of HF1551 overlaps the transcribed pseudogene GUSBP9. Several non-genic variants occur within long intergenic non-coding RNAs (lincRNA) coding regions (Additional file 7). Both these classes of genomic elements provide an additional tier of gene regulation, and contribute significantly to the transcriptional landscape of human cancers [134,135]. Several candidates also show interesting changes in their putative functions in cancer tissues. For example, we observe a MC on chromosome 2 (HF087) in a genomic region bearing a histone modification pattern characteristic of insulators in multiple different normal cell types, but the pattern changes to that of an enhancer in hepatocellular carcinoma.

Optical Mapping provides a global view of the cancer genome, free from biases introduced by cloning, amplification or hybridization, and discovers structural variation and mutation on a scale ranging from kilobases to megabases. Moreover, since the platform uses high-molecular weight DNA as analyte, the long-range context and connectivity of each variant is preserved, potentiating meaningful interpretation of candidate genes. However, Optical Mapping does not provide single-base resolution. Point mutations or indels spanning a few base pairs, such as the events frequently observed in CIC and FUBP1 genes in 1p/19q codeleted oligodendrogliomas, are below the lower limit of resolution and would remain undetected (unless they create or destroy a SwaI restriction site).

### Biological significance of candidates identified by optical mapping

The aim of this study is to generate new hypotheses for oligodendroglioma genetics, and as such, functional studies are beyond the scope of this paper. However, by surveying publicly available data on the candidates discerned by Optical Mapping, we can gain some insight into the roles they might play in malignant transformation.

Moving beyond the two tumors HF087 and HF1551, we wanted to take the candidate cancer genes and analyze them in the context of other genome-wide studies. Most somatic mutations in cancer cells arise due to genomic instability and do not contribute to tumorigenesis. However, mutations in genes that promote tumor development, so-called ‘driver’ mutations tend to be recurrent. To assess the extent of recurrence of our candidates across a large number of samples, we used the Catalog of Somatic Mutations in Cancer (COSMIC). The COSMIC database is a comprehensive archive of somatic mutations in human cancer, combining manually curated data from scientific literature and the output from the Cancer Genome Project [136,137]. 10 genes from our list of candidates had mutation frequencies (number of unique mutated samples divided by the total number of unique samples) greater than 10%, with the top hits being MYOF (19.1%), CECR2 (18.8%) and ZFYVE26 (18.7%). Mutation frequencies for all the candidate genes are listed in Table 2.

**Table 2:** Mutation frequencies of candidate cancer genes (COSMIC database)

**Gene symbol**	**No. of samples with mutations**	**No. of unique samples**	**Mutation frequency**
STMN2	3	517	0.580270793
EIF1	1	91	1.098901099
LRRN2	10	522	1.915708812
PAX7	15	748	2.005347594
PRKG1	21	545	3.853211009
ARHGAP10	9	229	3.930131004
PARK2	10	204	4.901960784
OSBPL3	11	206	5.339805825
APPL1	9	161	5.590062112
TCEB3	7	97	7.216494845
CCDC91	8	97	8.24742268
EFHC2	11	131	8.396946565
TACC2	30	312	9.615384615
NPAS3	13	114	11.40350877
CERS3/LASS3	12	104	11.53846154
SIPA1L3	12	102	11.76470588
DIAPH2	19	143	13.28671329
PLEKHM3	4	26	15.38461538
PHLDB2	17	108	15.74074074
ALMS1	28	165	16.96969697
ZFYVE26	35	187	18.71657754
CECR2	16	85	18.82352941
MYOF	22	115	19.13043478
LOC339166	0	0	-

Since cells can employ a number of mechanisms to compensate for loss or mutational inactivation of genes, a more direct way of assessing the functional role of a given candidate gene is to analyze changes in its pattern of expression between normal and disease states. Array based expression profiling of tumors HF087 and HF1551 was performed by Fine et. al. [138], and is publicly available through the NCBI GEO database [139], accession GSE4290. Differential expression analysis carried out using the EBarrays algorithm [140] shows that 5 genes (NPAS3, STMN2, ZFYVE26, PHLDB2 and PLEKHM3) from our list of 24 candidates is significantly up or down-regulated (p-value 1E-03 or less). A complete list of differentially expressed genes can be found in Additional file 8. To assay for functional effects in an even larger population of tumors, we queried for changes in expression of our candidate genes in REMBRANDT, a database of molecular data on brain tumors (National Cancer Institute, 2005, REMBRANDT home page https://caintegrator.nci.nih.gov/rembrandt/, accessed 13^th^August 2012). The results are reported for each gene as the number of oligodendroglioma samples in the database that are differential expression by at least two-fold (Additional file 9). All but 3 of our candidate genes were differentially expressed in at least 10 tumor samples. Congruent with the previous analysis, NPAS3, STMN2, ZFYVE26 and PHLDB2 are the most frequently deregulated candidate genes.

Finally, we asked what biological processes and pathways are significantly enriched or depleted in our list of candidates. This can identify fundamental cellular mechanisms that contribute to cancer development. As a whole, these candidate genes are enriched for proteins involved in cytoskeletal organization (p-value = 0.00223, after correcting for multiple testing, Ontologizer [141]). Our candidate genes are also significantly enriched for microRNA binding targets (p-values between 0.0144-0.0198 after correcting for multiple testing, WebGestalt [142]). Approximately half of the over-represented sites have been associated with binding of cancer-related microRNAs [143], underscoring the importance of post-transcriptional control of expression in oligodendroglioma.

While these results are not a direct indicator of aberrant function, this is a demonstration that Optical Mapping results can be expanded to clinical samples and used to create direct functional hypotheses.

## Conclusions

We have applied Optical Mapping to explore the genomic landscape of solid tumor oligodendroglioma. ~2100 discrete structural variants have been discovered, ranging in size from single base changes to loss of entire chromosomes. The structure of each alteration has been elucidated at sub-genic resolution, while retaining the long-range context of the event. 94 somatic mutations have been identified, 24 of which affect genes. These novel candidate cancer genes provide focused, testable hypotheses for follow-up functional investigation. We believe that Optical Mapping provides a comprehensive, high-resolution description of the complex and disperse genomes of solid tumors.

## Methods

### Selection of tumors

The tumors used in this study originated from the tissue bank at the Hermelin Brain Tumor Center/Department of Neurosurgery, Henry Ford Hospital (provided by Dr. Oliver Bogler). Freshly resected tumors were snap frozen in liquid nitrogen in the operating room. Samples were sectioned in a guillotine in frozen condition, and adjacent pieces prepared for Optical Mapping and for re-review by a neuropathologist.

The tumor samples selected for Optical Mapping had to meet two criteria. First, they needed to conform to the 1p/19q paradigm of treatment sensitivity. LOH status was assessed by quantitative PCR of microsatellite markers along chromosomes 1p and 19q (data not shown). Second, they needed to have a high proportion of cancer cells as opposed to normal cells. The percentage of tumor cells present in each biopsy was estimated by MIB-1 antibody staining of an adjacent section (data not shown). The MIB-1 antibody recognizes the Ki-67 antigen, which is a cell proliferation marker. For the most part, mitotic activity is absent in the adult brain, so the measurement of the Ki-67 cell proliferation marker can be used to judge tumor aggression and composition [144,145]. The two samples chosen for this project, HF087 and HF1551, satisfied both these criteria. Table 3 provides relevant clinical information for each tumor.

**Table 3:** Clinical information on the tumors analyzed by Optical Mapping

**Tumor sample**	**Histology**	**Histology code**	**LOH status**	**MIB Index (%)**	**Patient age**	**Patient sex**
HF087	Oligodendroglioma	O II	LOH	5-7%	65	Female
HF1551	Atypical Oligodendroglioma	O II	LOH	4-13%	30	Female

*O II:* Grade II; *LOH:* loss of heterozygosity.

### Extraction of high molecular weight DNA from solid tumor biopsies

The tumor was sectioned into 1–2 mm slices under sterile conditions in a cell culture hood. Each slice was treated with 0.8% type IV collagenase (Sigma-Aldrich, St. Louis, MO) in PBS (Phosphate buffered saline, Life Technologies, Carlsbad, CA) for 15 minutes at 37°C. The tumor tissue was mechanically disaggregated into a homogeneous suspension by repeated pipetting. The cells were pelleted by centrifugation at 1,000 RPM with a Beckman GS-6R centrifuge (Beckman Instruments, Fullerton, CA), and then resuspended in 1X HBS (Hanks Balanced Salts, Life Technologies, Carlsbad, CA) in order to lyse red blood cells. Cell debris and HBS were removed by centrifugation at 1,000 RPM. Finally, the pellet was rinsed three times with 35 mL of PBS, and resuspended in 0.5 mL of PBS.

A three layer Percoll gradient was employed to enrich for cancer cells, and minimize stromal contamination [146]. First, a 100% solution was made by using 9 parts Percoll (Sigma-Aldrich, St. Louis, MO) and 1 part 10X HBS, which was subsequently diluted with PBS to prepare 10%, 30%, and 50% solutions. The gradient was prepared by layering 2 mL of 50% Percoll, 2 mL of 30% Percoll, and 1 mL of 10% Percoll in a 15 mL Falcon tube. The single cell suspension was then carefully layered on top, and the gradient was spun at 1,000 RPM for 10 minutes. Studies have shown that cellular debris and non-viable cells are unable to penetrate the 30% layer, while lymphocytes pelleted at the bottom of the tube. The 30% layer, containing viable cells, was carefully removed, rinsed three times with 10 mL of PBS and then resuspended in PBS at a final concentration of 1X10^7^ cells/mL. Next, this cell suspension was mixed 1:1 (v/v) with 1.6% low gelling temperature agarose, poured into a mold and cooled to 4°C so that the agarose solidified to for gel inserts (each ~100 μL in volume).

The inserts were treated with 0.5 mg/mL proteinase K (Bioline USA, Taunton, MA), 100 mM EDTA pH 8.0 (Sigma-Aldrich, St. Louis, MO), 0.5% N-lauroylsarcosine (Sigma-Aldrich, St. Louis, MO) and incubated at 55°C overnight to lyse the tumor cells and degrade cellular proteins [45,147-150]. Embedding cells in agarose inserts eliminates shear induced breakage of genomic DNA molecules upon lysis [151].

Prior to use, the gel inserts were rinsed in TE twice for 1 hour and then a third time overnight to remove the detergent and excess EDTA. DNA was electrophoretically extracted by applying a cycle of 100 V for 30 seconds and -100 V for 6 seconds.

### Generation of single molecule optical maps

Optical Mapping surfaces were prepared as described earlier [152]. Briefly, acid-cleaned glass coverslips (22 × 22 mm, Fisher's Finest, Fisher Scientific) were treated with a mixture of N-trimethoxylsilylpropyl-N,N,N-trimethylammonium chloride and vinyltrimethoxysilane (Gelest, Morrisville, PA) rendering a positive charge to the surface. Genomic DNA, mixed with a sizing standard, was elongated *via c*apillary flow in a microfluidic device [66], and immobilized by electrostatic interactions with the positively charged surface, creating arrays of stretched, biochemically accessible substrates. The surface was then washed with TE (10 mM Tris–HCl, 1 mM EDTA, pH8.0) twice, equilibrated with digestion buffer (NEB buffer 3), then incubated with the restriction endonuclease SwaI (New England Biolabs, Beverly, MA), which cleaves the genomic DNA at its cognate site. Since the elongated DNA molecule is under slight tension, upon cleavage its ends relax, creating a 1–2 micron gap, readily detected by microscopy. The resulting restriction fragments remain adsorbed to the surface, aided by a polyacrylamide overlay, and hence retain their order creating, in essence, a barcode from each genomic DNA molecule. Restriction fragments were then stained with the DNA intercalating dye YOYO-1 (0.2 μM in β-mercaptoethanol/TE, Life Technologies, Carlsbad, CA) and imaged by automated fluorescence microscopy.

The images were collected on an Optical Mapping workstation, which consists of Zeiss 135M inverted microscope (Carl Zeiss, Thornwood, NY), illuminated by 488 nm argon ion laser (Spectra Physics, Santa Clara, CA) equipped with 63X oil immersion objective. Fully automated image acquisition software, referred to as Channel Collect [66], takes multiple overlapping images to span the entire length of each microchannel. The images were analyzed by custom machine vision software, called Pathfinder, which identifies DNA molecules on the surface and calculates the size of each restriction fragment based on integrated fluorescence intensity measurements relative to a sizing standard. Previous studies have shown that integrated fluorescence intensity scales with fragment mass, and is independent of stretch of the DNA molecule. The end result of these operations is the high throughput, massively parallel generation of single molecule ordered restriction maps, or optical maps, containing information about both the size and order of its restriction fragments.

### Pipeline for optical Map assembly and identification of structural variants

The analytical framework for assembly of optical maps is analogous to sequence assembly. First, our pipeline automatically aligned optical maps against a SwaI restriction map created *in silico* from the human genome reference sequence (NCBI build 35) *via* SOMA (Software for Optical Map Alignment) using gapped global pair wise alignment [56,67]. SOMA uses a scoring function that assigns penalties for differences in the optical map and the reference map, including missing or extra restriction sites, or differences in the size of the fragments that could represent insertions or deletions. The parameters of SOMA were set so that we are accurately aligning the molecule to the correct location, but loose enough for allow for a small number of differences that result from the mutations or polymorphisms present in the genome. The aligned maps were then partitioned into smaller bins (1 Mb windows spanning across each chromosome, with 500 kb overlap between adjacent windows) based on their location. The optical maps in each bin were assembled into optical consensus maps by a map assembler program, using a Bayesian inference algorithm [153]. Because some structural polymorphisms and mutations represent large-scale changes from the reference map, an iterative assembly process was used for the analysis of human data sets. The consensus map constructed in the previous step was used in place of the reference for seven more iterations of alignment and assembly, after which it was aligned to the reference sequence using SOMA. Using this strategy, Rmaps harboring major alterations that preclude alignment to the reference were gradually incorporated into the consensus map, extending it into regions that contain more complex rearrangements.

Lastly, the pipeline automatically performed analysis that tabulated structural variants using the final consensus map to the reference (derived from NCBI build 35 of the human genome [56]) and identified five classes of differences: missing cuts, extra cuts, insertions, deletions, and ‘other’ (multiple cut and/or size differences) across each cancer genome. Each of these differences, which are largely structural variants, has to satisfy certain statistical and empirical criteria. These parameters have been detailed in Teague et al. [56]. The only difference being the indel calling threshold, which was increased to a 13% change relative to the reference, with a 4.5 kb minimum.

Additionally, each structural variant was manually curated to ensure that the most conservative decision has been made at every locus. The genomic locations of the variants were converted to NCBI build 37 co-ordinates using the Batch Coordinate Conversion (liftover) tool from the University of California Santa Cruz Genome Browser [154] ( http://genome.ucsc.edu).

### Optical map coverage analysis

Variations in depth of coverage of optical maps aligned by SOMA across the genome can be used to detect copy number alterations. Intuitively, if a region of the tumor sample has increased (or decreased) copy number relative to the ‘normal’ reference genome, more (or less) maps will originate from it on an average. This is formalized as described. Pair-wise alignments of optical maps to an *in silico* reference were summarized by a single number (midpoint) representing location. These locations were modeled as realizations of a non-homogeneous Poisson process. The non-homogeneity arises from the fact that the likelihood of a map aligning to a genomic region depends on the density of restriction sites, and was accounted for using alignment data from a normal genome, which are used to define random intervals with counts that follow a negative binomial distribution. These counts were then modeled by a Hidden Markov Model, incorporating spatial dependence in the data and allowing more natural estimation of certain parameters [68,69].

### Affymetrix genome wide human SNP array 6.0

DNA was prepared for hybridization using the Blood and Cell Culture Kit (Qiagen, Valencia, CA), starting from frozen cells (HF087), or tumor tissue (HF1551), disaggregated into single cells as described previously. The HF087 cells were derived from the same slice used for Optical Mapping. However, since the same was not available for HF1551, a slice adjacent to the one used for mapping was used.

The DNA was digested with NspI and StyI restriction enzymes and ligated to adaptors that recognize the 4 bp overhangs. A generic primer that anneals to the adaptor sequence was then used to amplify adaptor-ligated DNA fragments, under PCR conditions optimized to preferentially amplify fragments in the 200 to 1,100 bp size range. The amplified DNA was then fragmented, labelled, and hybridized to a Genome-Wide Human SNP 6.0 Array (experiments were performed by the DNA Facility at the Carver College of Medicine, University of Iowa). Data analysis was performed using Genotyping Console 2.0 (Affymetrix, Santa Clara, CA). CNVs were called using either the Affymetrix algorithm (with default parameters) or five different algorithms (GLAD, Circular Binary Segmentation, Fused Lasso, Gaussian Model with Adaptive Penalty, Forward-Backward Fragment Annealing Segmentation) from CGHweb ( http://compbio.med.harvard.edu/CGHweb/) [155]. Only CNV calls made by two or more algorithms were considered for comparison.

### Parameters for comparing oligodendroglioma structural variants

#### ***To other optical mapping datasets***

Only variants of the same type were compared to each other, e.g.: MCs from HF087 were compared to MCs from lymphoblast cell line GM15510. Intersection ‘windows’ were set based on the type of OSA (100 bp for MCs, 4200 bp for ECs and 0 bp for INS, DEL and OTHER) and are reflective of the error processes inherent to each type of event.

#### ***To published SNPs and structural variants***

Published SNPs were compared against Optical Mapping cut differences using 100 bp or 3000 bp windows for MCs and ECs, respectively.

Structural variants from the latest (November 2010) release of the Database of Genomic Variants [156] were divided into two categories on the basis of their size. Events smaller than 3 kb were compared to ECs and MCs, since ~1/3^rd^ of indels that are below the lower limit of detection for Optical Mapping manifest themselves as cut differences [56]. Events larger than 3 kb were compared to INS, DEL and OTHER variants using a 0 bp intersection window.

### PCR validation

Template for PCR was prepared by whole genome amplification of tumor DNA using the REPLI-g Mini kit (Qiagen Inc., Valencia, CA) as per the protocol provided by the manufacturer. Pooled normal DNA from 6 individuals (Promega Corporation, Madison, WI) was used for control reactions. Primers were designed using freely available software Primer 3 Plus [157]. PCR reactions were performed using reagents from the Expand Long Template PCR System (Roche Applied Science, Indianapolis, IN) following the protocol supplied by the manufacturer. PCR reactions were digested with appropriate restriction enzymes to establish that the correct region had been amplified. The amplicon was then cloned in *E. coli* using the TOPO TA Cloning Kit (Invitrogen, Carlsbad, CA), plasmid DNA was purified using the Qiagen Plasmid Mini Kit (Qiagen Inc., Valencia, CA), and sequenced using Sanger biochemistry.

### Targeted assemblies on Williams-Beuren chromosomal region

The SwaI *in-silico* restriction map from the Williams-Beuren region on chromosome 14 was modified to reflect one of eight alterations: 4 possible inversions, each with unique start/end locations and spans (including the ‘canonical’ inversion), and 4 possible deletions, each with unique start/end locations and sizes (including the ‘canonical’ deletion). These modified *in-silico* maps were subjected to 8 rounds of iterative assembly, using the collection of HF1551 Rmaps, with the same parameters as the genome-wide assembly. The results were manually curated to rule out assembly errors.

### Ethics statement

This study was approved by the Institutional Review Board of the University of Wisconsin-Madison.

### Availability of supporting data

All structural variation calls and analysis are contained within the additional files.

## Abbreviations

OM: Optical mapping; Kb: kilobase pairs; EC: Extra cut; MC: Missing cut; INS: Insertion; DEL: Deletion; SNP: Single nucleotide polymorphism; CNV: Copy number variant; HMM: Hidden Markov Model; LOH: Loss of heterogeneity; CGH: Comparative genome hybridization; MHC: Major histocompatibility complex; DGV: Database of genomic variants; COSMIC: Catalog of somatic mutations in cancer; ENCODE: Encyclopedia of DNA elements.

## Competing interests

The authors declare that they have no competing interests.

## Authors’ contributions

MR carried out the experimental studies, analysis and manuscript writing. SG developed and applied new bioinformatic tools used for the study. SZ contributed to the data analysis. KP helped with the optical mapping and data interpretation. DS and MAN contributed new statistical tools. EE and CK assisted in the statistical analysis of structural variants. OB contributed samples and overall project guidance. DCS conceived the study, its design, manuscript writing and overall management of the project. All authors have read and approved the final manuscript.

## Supplementary Material

Additional file 1 **Basic attributes of oligodendroglioma datasets.** This spreadsheet describes the two optical maps in detail. Column A lists specific characteristics associated with each step of constructing the map. Columns B and C lists its values for tumor HF087 (column B) and HF1551 (column C). Click here for file

Additional file 2 **Overlap (counts) between oligodendroglioma structural variants and genes, segmental duplications, SNPs, and variants reported by other investigators.** This table lists counts of structural variants from oligodendroglioma that intersect with genes, segmental duplications, SNPs, and events from the Database of Genomic Variants (DGV). Column A specifies the genomic element; columns B and H indicate total counts for HF087 and HF1551 respectively; Columns C-G and I-M shows overlap counts by variant class. Variants from the DGV are divided based on size into those over 3 kb and those under then 3 kb, then further by study. Variants less than 3 kb are compared to optical map EC and MCs, while those over 3 kb are compared to INS, DEL and OTHER. Click here for file

Additional file 3 **Detailed description of intersections between oligodendroglioma structural variants and genes, SNPs, variants from the Database of Genomic Variants and other normal human optical maps.** This spreadsheet provides a detailed breakdown of the overlap between Each row in the spreadsheet shows an optical map difference (column A), it’s location (columns B-D), and genes (column E), variants from the Database of Genomic Variants (column F, G), snip-SNPs (column H), and structural variants from other Optical Mapping datasets (columns I-N) that overlap with it. The number is parenthesis after each column header indicates the intersection window. Click here for file

Additional file 4 **Experimental validation of oligodendroglioma ECs and MCs by SNP array.** This table lists cut differences found in HF087 and HF1551 (column A), their location (columns B-D), type (column E), the SNP genotype corresponding to it (column F) and whether it agrees with the optical map (column G). Click here for file

Additional file 5 **Experimental validation of oligodendroglioma indels.** This table lists indels found in HF087 and HF1551 (column A), their location (columns B-D) and whether it is validated by a given copy number algorithm (columns E-J). Click here for file

Additional file 6 **Oligodendroglioma structural variants and their intersection (counts) with variants detected in six other normal human optical maps.** This table lists counts of structural variants from oligodendroglioma that intersect with variants found in other normal human genomes that have been analyzed by Optical Mapping. Column A specifies the genomic element; columns B and H indicate total counts for HF087 and HF1551 respectively; Columns C-G and I-M shows overlap counts by variant class. Only variants of the same category are included in the comparison. Click here for file

Additional file 7 **Non-genic candidates found in oligodendroglioma and functional elements from ENCODE that intersect them.** Column A lists identifiers for candidate loci that do not occur within a gene, their locations (columns B-D), overlapping transcripts found by GENCODE (column E), and predicted genomic state in different cell types (columns F-N). The cell types in red font are cancer cell lines. Different genomic states are color-coded as per ENCODE website, and is detailed in the key. Click here for file

Additional file 8 **Differentially expressed genes in GEO dataset GSE4290, analyzed by Ebarrays, p = 1E-03.** Entrez gene identifer (column A), gene symbol (column B), gene name (column C), cellular location (column D) and molecular function (column E) of all differentially expressed genes in GEO dataset GSE4290. Click here for file

Additional file 9 **Number of oligodendrogioma samples in REMBRANDT database where a given candidate gene is up or down regulated by at least 2 fold.** This table lists candidate genes identified through Optical Mapping (column A), and the number of oligodendroglioma samples in the REMBRANDT database where that gene is up or down regulated by at least two fold (column B). Click here for file
